# Effect of time to palliative epilepsy surgery on outcomes

**DOI:** 10.1002/epi.70379

**Published:** 2026-08-03

**Authors:** Robert M. Crutcher, David E. Horvat, Avery R. Caraway, Kristen H. Arredondo, Taylor J. Abel, Nitin Agarwal, Allyson L. Alexander, Dallas Michael Armstrong, Kurtis I. Auguste, Danilo Bernardo, Jeffrey Bolton, David Bonda, Dewi Frances Depositario‐Cabacar, Krista Eschbach, William D. Gaillard, Aran Groves, Jason S. Hauptman, Pradeep K. Javarayee, Cemal Karakas, Andrew T. Knox, Jenny J. Lin, Patricia E. McGoldrick, Derryl J. Miller, Edward John Novotny, Adam P. Ostendorf, Pilar D. Pichon, Shilpa B. Reddy, Debopam Samanta, Rani K. Singh, Janelle L. Wagner, Steven M. Wolf, Lily C. Wong‐Kisiel, Fernando N. Galan, M. Scott Perry, Michael A. Ciliberto

**Affiliations:** ^1^ Department of Child and Adolescent Neurology Nemours Children's Health Jacksonville Florida USA; ^2^ Department of Neurology Alexander T. Augusta Military Medical Center Ft. Belvoir Virginia USA; ^3^ Department of Neurology Jane and John Justin Institute for Mind Health, Cook Children's Medical Center Fort Worth Texas USA; ^4^ Department of Neurology, Dell Medical School University of Texas at Austin Austin Texas USA; ^5^ Department of Neurological Surgery School of Medicine, University of Pittsburgh Pittsburgh Pennsylvania USA; ^6^ Division of Pediatric Epilepsy Minnesota Epilepsy Group Roseville Minnesota USA; ^7^ Division of Pediatric Neurosurgery, Children's Hospital Colorado; Department of Neurosurgery University of Colorado Anschutz School of Medicine Aurora Colorado USA; ^8^ Department of Pediatrics and Department of Neurology University of Texas Southwestern Medical Center Dallas Texas USA; ^9^ Department of Neurosurgery University of California, San Francisco Weill Institute for Neurosciences, Benioff Children's Hospital San Francisco California USA; ^10^ Department of Neurology, Division of Epilepsy University of California, San Francisco San Francisco California USA; ^11^ Department of Neurology Harvard Medical School Boston Massachusetts USA; ^12^ Department of Neurology Boston Children's Hospital Boston Massachusetts USA; ^13^ Department of Neurosurgery Cedars‐Sinai Los Angeles California USA; ^14^ Department of Pediatrics Guerin Children's, Cedars‐Sinai Medical Center Los Angeles California USA; ^15^ Department of Neurology, Center for Neuroscience Children's National Hospital, George Washington University School of Medicine Washington District of Columbia USA; ^16^ Section of Child Neurology, Department of Pediatrics University of Colorado Anschutz Medical Campus Children's Hospital Colorado Aurora Colorado USA; ^17^ Division of Neurology OHSU Doernbecher Children's Hospital Portland Oregon USA; ^18^ Department of Neurosurgery Phoenix Children's Hospital Phoenix Arizona USA; ^19^ Department of Neurology Medical College of Wisconsin Milwaukee Wisconsin USA; ^20^ Department of Neurology University of Wisconsin School of Medicine and Public Health Madison Wisconsin USA; ^21^ Department of Pediatric Neurology Children's Healthcare of Atlanta Atlanta Georgia USA; ^22^ Department of Neurosurgery St. Luke's/Roosevelt Hospital Center and Beth Israel Medical Center New York New York USA; ^23^ Department of Neurology Specialty Care Brentwood Tennessee USA; ^24^ Division of Pediatric Neurology, Department of Neurology Neurosciences, Seattle Children's Hospital, University of Washington Seattle Washington USA; ^25^ Department of Pediatrics Neurosciences, Seattle Children's Hospital, University of Washington Seattle Washington USA; ^26^ Department of Neurology Nationwide Children's Hospital and Ohio State University Columbus Ohio USA; ^27^ Department of Neurology Children's Hospital of Orange County Orange California USA; ^28^ Department of Neurology Monroe Carell Jr. Children's Hospital at Vanderbilt Nashville Tennessee USA; ^29^ Child Neurology Division, Department of Pediatrics University of Arkansas for Medical Sciences Little Rock Arkansas USA; ^30^ Department of Neurology Atrium Health, Levine Children's Hospital Charlotte Charlotte North Carolina USA; ^31^ College of Nursing, Department of Pediatrics, and Comprehensive Epilepsy Center Medical University of South Carolina Charleston South Carolina USA; ^32^ Department of Neurology Mayo Clinic Rochester Minnesota USA; ^33^ Child Neurology, Department of Pediatrics University of Iowa Iowa City Iowa USA

**Keywords:** epilepsy, palliative, refractory, surgery

## Abstract

**Objective:**

Epilepsy duration is a modifiable risk factor in the outcome of definitive epilepsy surgery; however, an analogous effect in palliative procedures has not been shown. We reviewed the Pediatric Epilepsy Surgery Database data for an association between epilepsy duration and seizure reduction in palliative procedures.

**Methods:**

Patients enrolled between January 2018 and April 2025 who underwent their first epilepsy surgery with palliative intent with 6 months of follow‐up were included. Procedures included neuromodulation, corpus callosotomy, hemispherotomy, lesionectomy, and lobectomy where surgical intent was not seizure freedom. Outcomes of seizure freedom, 90% seizure reduction, and 50% seizure reduction were considered at 6–12 months and >12 months from surgery. Duration from epilepsy onset to surgery was compared for patients above and below each outcome threshold at each time point. Logistic regression analysis for the association between epilepsy duration and seizure reduction adjusted for potential confounders including procedure type, etiology, and other clinical factors. Logistic regression analysis was performed on the overall cohort and subgroups of patients with each procedure.

**Results:**

A total of 588 patients were included. Initial univariate analysis suggested that epilepsy duration at time of surgery was significantly associated with seizure freedom and 90% seizure reduction at both 6–12 months and >12 months. After adjusting for confounders, only seizure freedom at >12 months was significantly associated with duration of epilepsy. When individual procedures were considered, only lobectomy was sensitive to duration of epilepsy in multivariate analysis, with significant impacts on >50% and >90% seizure reduction at >12 months. Lesional epilepsy predicted seizure freedom at >12 months. Neuromodulation and corpus callosotomy were less likely to achieve seizure reduction than other procedures.

**Significance:**

We did not find an association between early epilepsy surgery and seizure reduction. This reflects the heterogeneity of our population, including different types of surgical procedures and lesional and nonlesional epilepsies.

**Key points**

- There was no association between epilepsy duration and ≥50% or >90% seizure reduction in our cohort after adjusting for confounders.
- There was an association between epilepsy duration and ≥50% and >90% seizure reduction in the subgroup of patients who received lobectomy.
- More than 50% of resective procedures in our cohort achieved seizure freedom, raising concern that some of these surgeries should be classified as curative rather than palliative.

## 1 INTRODUCTION

Epilepsy is common, with a prevalence of .5%–1% in the pediatric population.^1^ Approximately 30% of pediatric patients develop drug‐resistant epilepsy (DRE), defined by the International League Against Epilepsy (ILAE) as the failure to achieve seizure freedom with two appropriately chosen and dosed antiseizure medicines (ASMs).^2^, ^3^ Select patients with DRE undergo surgical resection or ablation with the goal of stopping seizures entirely.^2^ However, many patients may not attain complete seizure freedom with epilepsy surgery due to poor seizure localization, multiple foci, or lesions in eloquent cortex not amenable to resection.^4^, ^5^ These patients may be offered one of many surgical procedures with the goal of mitigating severity of disease. We refer hereafter to “curative” epilepsy surgery where the expectation of the procedure is seizure freedom, and epilepsy surgery with “palliative” intent where the expectation is not seizure freedom, but rather reduction in seizures or cessation of particular seizure types.^4^, ^5^ Traditional palliative procedures include neuromodulation and corpus callosotomy; however, hemispherotomy, lesionectomy, or lobectomy can be palliative depending on the anticipated surgical outcome.^4^, ^5^

Although ILAE guidelines recommend referral for surgical evaluation as soon as the diagnosis of DRE is established, patients face significant delays between diagnosis, referral, and surgery.^6^, ^7^ The average time between epilepsy diagnosis and surgery is approximately 5 years in children and approximately 20 years in adults.^6^ Several explanations have been offered for these delays, including a lack of physician knowledge of surgical indications, an assumption that referral equals commitment to undergoing a surgical procedure, and social determinants of health, including insurance, geographic location, and cultural acceptance/understanding of surgery.^5^, ^7^ Compared to candidates for curative surgery, epilepsy surgery with palliative intent patients wait 2–3 years longer and trial two more ASMs prior to referral to a surgical center.^8^ Hesitation in palliative procedures may be compounded by a perceived smaller benefit and the stigma of the term “palliative” itself.^4^, ^9^

The literature is clear that a shorter duration of epilepsy at time of curative surgery improves outcomes. A 2019 meta‐analysis by Bjellvi et al. found that shorter epilepsy duration at the 2‐, 5‐, 10‐, and 20‐year thresholds consistently improved the chance of seizure freedom for all resective surgeries.^10^ This finding was replicated by Wagystl et al., who found improved outcomes with shorter epilepsy duration in focal cortical dysplasia.^11^ Lamberink et al. also found the same association with any structural cause.^12^

There is a paucity of data on the association between epilepsy duration and seizure reduction in palliative procedures. With respect to the surgical option of neuromodulation, vagal nerve stimulation (VNS) is the oldest and most well studied. Zhu et al. found that a 50% seizure reduction was significantly associated with duration of epilepsy less than 12.5 years at VNS implantation, and Renfroe and Wheless and Crutcher et al. both found a similar association with epilepsy duration less than 5 years.^13^, ^14^, ^15^ There are no studies in the literature describing an association between epilepsy duration and outcome with either responsive neurostimulation (RNS) or deep brain stimulation (DBS). For corpus callosotomy, a meta‐analysis by Chan et al. found that epilepsy duration <15 years was associated with seizure freedom.^16^ There are no studies examining the relationship of epilepsy duration to seizure frequency reduction outcomes after palliative resective procedures, and there are no large, multicenter studies on this subject that systematically include a range of procedures performed with palliative intent.

We conducted a retrospective cohort study of patients undergoing epilepsy surgery with palliative intent using the Pediatric Epilepsy Research Consortium (PERC) surgery database. We hypothesized that pediatric patients who have a shorter duration to epilepsy with palliative intent surgery have a higher probability of achieving a meaningful reduction in seizure frequency compared to those patients whose surgeries are delayed. This question is important to patients with newly diagnosed DRE weighing options of epilepsy surgery with palliative intent against additional ASM trials or dietary therapy.

## 2 MATERIALS AND METHODS

### 2.1 Data collection

The Pediatric Epilepsy Surgery Database of PERC is a collaboration of more than 30 pediatric epilepsy centers in the United States prospectively enrolling children 0–18 years old referred for epilepsy surgery evaluation since 2018. Enrolling institutions obtain institutional review board approval at their site. Detailed methods of data collection have been described previously.^17^ The data that support the findings of this study are available from the corresponding author upon reasonable request.

Patients enrolled between January 2018 and April 2025 who completed an initial epilepsy surgery were included. We included only surgeries classified as palliative after a multidisciplinary epilepsy surgery conference determined that seizure freedom was not an expected result of surgery. This decision was made by the enrolling center, prior to the surgery being performed. We excluded patients who did not have at least 6 months of postsurgical follow‐up, or who were missing data necessary to calculate the duration between epilepsy onset and surgery. Surgical success is input by the recording center at time points determined by the center. A small number of patients underwent multiple procedures in the same operation (e.g., hemispherotomy in addition to neuromodulation); for these patients, a single procedure type was assigned in order of most to least invasive: hemispherotomy, lobectomy, lesionectomy, corpus callosotomy, and neuromodulation. PERC does not enroll patients who are only evaluated for VNS, but some patients enrolled in the epilepsy surgery database ultimately underwent VNS surgery as their initial procedure and were included in our cohort.

For all patients in our cohort, we collected clinical data from the PERC database including age at epilepsy onset, age at surgery, type of procedure performed, number of procedure stages, frequency of seizures, types of seizures, number of seizure types, number of ASMs failed, etiology (e.g., structural, genetic), abnormal MRI findings, and abnormal neurologic exam. We also collected demographic information including race, ethnicity, sex, type of insurance, and distance from surgical center. Duration from epilepsy onset to surgery was calculated from the relevant time points as collected above and measured in years. The rest of our data were categorical variables with groups listed in Table 1. Seizure type was categorized as focal, generalized, or unknown according to ILAE definitions.^18^

**TABLE 1:** Clinical characteristics of our cohort prior to undergoing initial epilepsy surgery (*N* = 513).

Characteristic	*n* (%)
MRI result	
Normal	202 (40%)
Abnormal	310 (60%)
Neurological exam	
Normal	172 (34%)
Abnormal	339 (66%)
Primary seizure type	
Focal onset	295 (58%)
Generalized onset	193 (38%)
Unknown/subclinical onset	24 (5%)
Multiple seizures	350 (68%)
Surgery details	
1 stage	374 (72.9%)
2 stage	139 (27.1%)
Years with epilepsy [from onset to surgery]	
Median	6.1
Minimum, maximum	.1, 19.3
Mean age at onset (SD)	4.2 (4.2)
Mean age at phase 1 (SD)	10.3 (5.2)
Mean age at surgery (SD)	11.2 (5.1)
Mean number of failed ASMs (SD)	4.3 (2.9)

Abbreviations: ASM, antiseizure medicine; MRI, magnetic resonance imaging.

Outcomes of surgical procedure were gathered from predefined inputs to the epilepsy surgery database and measured as binary variables indicating whether a patient achieved the following thresholds of seizure reduction: ≥50% seizure reduction (vs. <50% reduction), >90% seizure reduction (vs. ≤90% reduction), and seizure freedom. These outcomes were cumulative in the sense that a patient who achieved seizure freedom was also counted in the >90% seizure reduction and >50% seizure reduction groups. Each outcome was assessed at two time periods: 6–12 months after surgery and >12 months after surgery. If multiple data entries existed in a given window, the most recent data were considered.

### 2.2 Statistical analysis

Statistical analysis was completed using R version 4.4.3.^19^ Duration of epilepsy at time of surgery was nonnormally distributed as evidenced by visual inspection of the histogram and normal Q–Q plot, the Shapiro–Wilk normality test (*p* < .001), and the Kolmogorov–Smirnov test (*p* < .001). Therefore, the median was used in lieu of the mean to describe this variable, and the Kruskal–Wallis test was used in lieu of analysis of variance to assess significant differences among various subpopulations. We used the standard threshold for statistical significance *α* = .05.

For each follow up window (6–12 months and >12 months) and each threshold of seizure reduction (seizure freedom, >90% seizure reduction, and ≥50% seizure reduction), we constructed a binary logistic regression model to analyze the impact of epilepsy duration at time of surgery on the probability of attaining the threshold seizure reduction. We adjusted for covariates that showed significant associations with epilepsy duration in univariate analysis. Odds ratios and *p*‐values were calculated from the maximum likelihood estimation of beta coefficients. We constructed additional logistic regression models for specific subpopulations including groups of patients with each procedure type, including the same covariates.

## 3 RESULTS

There were a total of 752 patients enrolled in the PERC database during the specified enrollment period who underwent first epilepsy surgery with palliative intent. Seventy‐five patients were excluded due to missing data on epilepsy duration at time of surgery, and 164 were excluded due to lack of follow‐up data beyond 6 months. A total of 513 patients were included in the final cohort. The median duration of follow‐up was 21 months, with a range of 6–72 months. A total of 329 patients had follow‐up data in the 6–12‐month window after surgery, and 384 patients had follow‐up data available after 12 months. We note that there were some patients who did not have follow‐up data prior to 12 months.

Clinical characteristics for our population are shown in Table 1. Our population was 56% (*n* = 288 patients) male, 75% (*n* = 384) White, 12% (*n* = 65) non‐White, 2% (*n* = 13) multirace, and 10% (*n* = 51) unknown race. Twenty percent (*n* = 101) of patients were Hispanic or Latino. Fifty percent (*n* = 254) lived within 50 miles of the surgical center, and 48% (*n* = 246) had public insurance. The median duration from epilepsy onset to surgery was 6.1 years, with a duration of .1–19.3 years.

Table 2 shows the composition of surgical procedures and postsurgical outcomes in our cohort. In order of decreasing frequency, procedures performed included 223 (43%) neuromodulation, 106 (21%) corpus callosotomy, 63 (12%) lesionectomy, 47 (9%) lobectomy, 35 (7%) hemispherotomy, and 32 (6%) thermal ablation. For patients who received neuromodulation devices, 133 (26% of the cohort) received VNS, 73 (14%) received RNS, and 16 (3%) received DBS. Seven patients underwent more than one procedure at the time of initial surgery. At the most recent follow‐up available, 63% (*n* = 322) of patients achieved ≥50% seizure reduction, 38% (*n* = 196) achieved >90% seizure reduction, and 25% (*n* = 126) achieved seizure freedom. Twenty‐seven (5.3%) patients underwent subsequent epilepsy surgery, and there were five (1.0%) deaths.

**TABLE 2:** Procedures and postsurgical outcomes of patients in our cohort.

Procedure	*n* (%)						
	Total^a^	Seizure‐free	>90% reduction^b^	≥50% reduction^b^	<50% reduction	Additional surgery	Death
Callosotomy	106 (20.7)	13 (12.3)	26 (24.5)	64 (60.4)	34 (32.1)	5 (4.7)	3 (2.8)
Hemispherotomy	35 (6.8)	22 (62.9)	26 (74.3)	31 (88.6)	3 (8.6)	1 (2.9)	0 (.0)
Lesionectomy	63 (12.3)	32 (50.8)	45 (71.4)	52 (82.5)	6 (9.5)	5 (7.9)	0 (.0)
Lobectomy	47 (9.2)	25 (53.2)	29 (61.7)	35 (74.5)	9 (19.1)	3 (6.4)	0 (.0)
Thermal ablation	32 (6.2)	16 (50.0)	23 (71.9)	24 (75.0)	4 (12.5)	4 (12.5)	0 (.0)
Neuromodulation	223 (43.5)	15 (6.7)	45 (20.2)	111 (49.8)	101 (45.3)	9 (4.0)	2 (.9)
DBS	16 (3.1)	1 (6.3)	1 (6.3)	8 (50.0)	8 (50.0)	0 (.0)	0 (.0)
RNS	73 (14.2)	9 (12.3)	20 (27.4)	42 (57.5)	27 (37.0)	2 (2.7)	2 (2.7)
VNS	133 (25.9)	5 (3.8)	23 (17.3)	60 (45.1)	66 (49.6)	7 (5.3)	0 (.0)
Multiple/other	7 (1.4)	3 (42.9)	3 (42.9)	5 (71.4)	2 (28.6)	0 (.0)	0 (.0)
Total	513 (100)	126 (24.6)	197 (38.4)	322 (62.8)	159 (31.0)	27 (5.3)	5 (1.0)

*Note*: Outcome data in this table reflect the most recent follow‐up available. Abbreviations: DBS, deep brain stimulation; RNS, responsive neurostimulation; VNS, vagal nerve stimulation.

^a^ Percentages in the Total column reflect the proportion of the total cohort undergoing each procedure. Percentages in remaining columns reflect the proportion of patients for each procedure that achieved a given outcome.

^b^ The >50% and >90% seizure reduction columns are cumulative.

We assessed each of the above clinical and demographic factors for a significant univariate association with the duration between epilepsy onset and surgery. There were significant differences (*p* < .01) in duration of epilepsy for many procedure types, with hemispherotomy (median = 3.5, interquartile range [IQR] = 1.7–5.9 years) and lesionectomy (median = 3.8, IQR = 1.7–6.7 years) having shorter median durations than other procedures, and neuromodulation (median = 7.2, IQR = 4.3–10.4 years) and corpus callosotomy (median = 7.7, IQR = 4.1–12.4 years) having longer durations than other procedures. Other variables that were associated with shorter time to surgery included the presence of a lesion on MRI, a normal neurologic exam, and seizure semiology (focal vs. generalized), and we include these variables in our multivariable analysis below. Notably, demographic factors including race, sex, type of insurance, and distance from epilepsy center did not have a significant impact on time to surgery in our cohort.

In univariate analysis, shorter epilepsy duration is associated with seizure reduction for nearly all outcome measures (Table 3). In the 6–12‐month time window, median duration of epilepsy was shorter for those achieving seizure freedom (3.9 vs. 7.2 years, *p* < .01) and >90% seizure reduction (5.1 vs. 7.3 years, *p* < .01). At 12+‐month follow‐up, all thresholds of seizure reduction were associated with epilepsy duration: seizure freedom (4.0 vs. 7.4 years, *p* < .01), >90% seizure reduction (4.6 vs. 7.6 years, *p* < .01), and ≥50% seizure reduction (5.7 vs. 7.5 years, *p* = .01).

**TABLE 3:** Associations between epilepsy duration and seizure reduction.

Epilepsy onset to surgery (years)	Median (IQR)	*p*
6–12 month outcomes, *n* = 329		
Seizure freedom (78)	3.9 (1.9–8.4)	<.01
No seizure freedom (251)	7.2 (4.1–10.6)	
>90% reduction (122)	5.1 (2.6–9.3)	<.01
Less reduction (207)	7.3 (4.2–10.7)	
≥50% reduction (212)	6.3 (3.1–9.8)	.25
Less reduction (117)	6.9 (4.0–10.6)	
12+ month outcomes, *n* = 384		
Seizure freedom (91)	4.0 (2.2–7.5)	<.01
No seizure freedom (293)	7.4 (3.8–11.0)	
>90% reduction (148)	4.6 (2.5–9.0)	<.01
Less reduction (236)	7.6 (4.0–11.1)	
≥50% reduction (241)	5.7 (2.9–9.9)	.01
Less reduction (143)	7.5 (4.0–10.8)	

*Note*: Probability values were computed using the Kruskal–Wallis test. Abbreviation: IQR, interquartile range.

In multivariable analysis, we examined the relationship between epilepsy duration and seizure reduction outcomes while adjusting for all variables that showed a significant association with epilepsy duration. After adjusting for these covariates (Table 4), only the relationship between epilepsy duration and seizure freedom at 12+ months remained statistically significant (odds ratio [OR] = .92, *p* = .02). The procedure type was a significant confounder; both corpus callosotomy and neuromodulation procedures were associated with decreased odds of seizure freedom and >90% seizure reduction at both 6–12 months and 12+ months. Neuromodulation procedures were also associated with decreased odds of ≥50% seizure reduction at 12+ months. Of note, the sample size of patients undergoing DBS was too small for analysis in the models predicting seizure freedom and >90% seizure reduction, as fitted probabilities approached 0 or 1. No other variables were significantly associated with any threshold of seizure reduction at 6–12‐month follow‐up. The presence of a lesion on magnetic resonance imaging (MRI) was a favorable indicator for achieving seizure freedom at >12 months (OR = 2.14, *p* = .032), whereas an abnormal neurologic exam was associated with decreased odds of >90% seizure reduction at 12+ months (OR = .54, *p* = .039).

**TABLE 4:** Multivariable logistic regression analysis for each threshold of seizure reduction at 12+ months.

Variable	12+ months		
	Seizure freedom	>90% reduction	≥50% reduction
Epilepsy onset to surgery (1‐year increase)	.92 [.86–.98]*	.96 [.91–1.01]	.97 [.92–1.02]
Lesion present (vs. not)	2.14 [1.08–4.36]*	1.32 [.76–2.30]	.83 [.50–1.36]
Abnormal neurological exam (vs. normal)	.52 [.27–1.00]	.54 [.30–.97]*	.81 [.47–1.38]
3+ ASMs (vs. 0–2)	1.24 [.68–2.29]	.98 [.58–1.67]	.97 [.60–1.58]
Generalized seizure (vs. focal)	1.23 [.59–2.61]	.94 [.52–1.74]	.91 [.53–1.55]
Unknown seizure (vs. focal)	1.31 [.38–4.37]	1.70 [.58–1.67]	3.09 [.93–14.10]
>1 seizure type (vs. 1)	1.55 [.84–2.92]	1.08 [.63–1.86]	1.14 [.68–1.89]
Corpus callosotomy (vs. other)	.25 [.08–.78]*	.19 [.06–.56]*	.45 [.14–1.33]
Hemispherotomy (vs. other)	1.36 [.41–4.57]	.80 [.22–2.85]	1.49 [.35–6.84]
Lobectomy (vs. other)	1.11 [.39–3.17]	.49 [.15–1.47]	.77 [.22–2.60]
Lesionectomy (vs. other)	.65 [.25–1.72]	.70 [.23–2.02]	1.19 [.35–3.90]
DBS (vs. other)	–	–	.18 [.03–.87]*
RNS (vs. other)	.16 [.05–.48]*	.13 [.04–.36]*	.35 [.11–.96]*
VNS (vs. other)	.05 [.01–.17]*	.07 [.02–.20]*	.20 [.07–.53]*

*Note*: Each column represents a separate model for a specified binary outcome (e.g., ≥50% vs. <50% seizure reduction). Each cell contains the odds ratio point estimate [95% confidence interval] representing each covariate's impact on the odds of a patient achieving a specified level of seizure reduction. The sample size of DBS patients was too small to calculate an odds ratio for seizure freedom and >90% seizure reduction. There was no significant association between epilepsy duration and any threshold of seizure reduction in the 6–12‐month follow‐up period. Abbreviations: ASM, antiseizure medicine; DBS, deep brain stimulation; RNS, responsive neurostimulation; VNS, vagal nerve stimulation.

*p < .05.

Because the goal of epilepsy surgery with palliative intent varies between different procedures, we repeated the same multivariable analysis among subgroups of patients undergoing each procedure (Table 5). The sample size of patients undergoing thermal ablation at 6–12 months and the sample size of patients undergoing DBS were too small for analysis, as fitted probabilities approached 0 or 1. Among patients undergoing lobectomy, at 12+ months, epilepsy duration was significantly associated with >90% seizure reduction (OR = .77, *p* = .02) and ≥ 50% seizure reduction (OR = .75, *p* = .03). There was no one procedure for which epilepsy duration was significantly associated with seizure freedom at 12+ months. However, the procedures that showed the strongest effect size for this outcome were resective procedures, in particular lobectomy (OR = .83, 95% confidence interval [CI] = .66–1.00, *p* = .07) and thermal ablation (OR = .79, 95% CI = .61–1.01, *p* = .06). By contrast, in neuromodulation procedures, there was no association between epilepsy duration and seizure freedom, with ORs clustered around 1. There were no other statistically significant associations between epilepsy duration and any threshold of seizure reduction for any procedure or time period.

**TABLE 5:** Subgroup analysis of the effect of epilepsy duration on surgical outcomes for each procedure.

Epilepsy duration (1‐year increment)	12+ months		
Procedure	Seizure freedom	>90% reduction	≥50% reduction
Hemispherectomy	.88 [.68, 1.07]	.92 [.73, 1.13]	.85 [.65, 1.11]
Corpus callosotomy	.92 [.78, 1.05]	.95 [.85, 1.07]	.94 [.85, 1.04]
Lesionectomy	1.02 [.86, 1.21]	1.07 [.89, 1.31]	1.09 [.88, 1.45]
Lobectomy	.83 [.66, 1.00]	.77 [.60, .94]	.75 [.56, .94]
Thermal ablation	.79 [.61, 1.01]	.88 [.64, 1.15]	.88 [.64, 1.15]
DBS	N/A	N/A	N/A
RNS	1.04 [.79, 1.37]	.98 [.08, 4.00]	1.03 [.30, 1.19]
VNS	1.01 [.76, 1.30]	1.01 [.87, 1.16]	.98 [.88, 1.09]

*Note*: We repeated multivariable logistic regression analysis (see Table 4) isolating individual procedures. Data shown are odds ratios [95% confidence intervals] for the effect of epilepsy duration on achieving a specified threshold of seizure reduction for the specified procedure at 12+ months of follow‐up. The number of DBS patients was insufficient to calculate odds ratios. There were no statistically significant associations at 6–12‐month follow‐up.

Abbreviations: DBS, deep brain stimulation; N/A, not applicable; RNS, responsive neurostimulation; VNS, vagal nerve stimulation.

## 4 DISCUSSION

In our retrospective analysis of a cohort of patients undergoing epilepsy surgeries with palliative intent, we did not find that epilepsy duration at the time of surgery was significantly associated with either ≥50% seizure reduction or >90% seizure reduction, after adjusting for confounders. A shorter duration of epilepsy was significantly associated with seizure freedom more than 12 months after surgery. When considering each procedure individually, a shorter duration of epilepsy was associated with ≥50% and >90% seizure reduction at >12 months after surgery for patients undergoing lobectomy, but not for other resective procedures, corpus callosotomy, or neuromodulation.

The finding that shorter epilepsy duration is associated with seizure freedom in our cohort is interesting, as by definition seizure freedom should not be an expectation of epilepsy surgery with palliative intent. When we evaluate individual procedures (Table 5), this association appears to be driven by resections, including lobectomy and thermal ablation, and not by neuromodulation or corpus callosotomy. The relatively high rates of seizure freedom seen in 50%–60% of resections in our cohort indicate that either these procedures outperformed presurgical expectations or that some of these procedures should have been classified as curative procedures.

Our data raise a broader issue about the definition of epilepsy surgery with palliative intent when applied to resections. As described in Materials and Methods, procedures were classified as palliative by the enrolling center in presurgical evaluation, using the criterion that seizure freedom was not an expected outcome. It is possible, however, that differences in provider judgment could lead to poor interrater reliability between centers in determining which surgeries are palliative. It has been documented that seizure freedom can occur with a single limited surgery despite bilateral seizure onset, indicating the possibility that network disruption is involved in outcome.^20^ There are also data showing that patients with generalized electroencephalographic findings and/or clinical semiologies can achieve seizure freedom with resective surgery if there is an underlying lesion.^21^, ^22^, ^23^ Our study suggests that patients who are potential candidates for resection should be expeditiously referred for surgical evaluation even if the odds of achieving seizure freedom are perceived to be suboptimal. Future work should create a more standardized definition, and a more appropriate term than “palliative,” to properly describe this group of resective surgeries.

For most procedures and thresholds of seizure reduction, we did not find that a shorter duration from epilepsy onset to surgery conferred a higher chance of achieving a meaningful reduction in seizure frequency. We caution that these negative findings do not imply that there is no harm in delaying evaluation for epilepsy surgery. More than 60% of patients in our cohort achieved ≥50% seizure reduction and nearly 25% achieved seizure freedom, metrics that typically outperform those of additional medication trials beyond two ASMs.^24^ Rather, our findings indicate that the surgical outcomes of epilepsy surgeries with palliative intent are more strongly influenced by clinical factors such as the type of procedure performed and presence of a lesion than whether the procedure is performed earlier or later in the course of epilepsy. Second‐order impacts of seizure reduction following epilepsy surgery with palliative intent include improved cognition and reduced risk of sudden unexpected death in epilepsy, and the duration from epilepsy to surgery may be an important effect modifier of these outcomes. However, our study was not designed to evaluate these measures.

Our study has several limitations. As discussed above, there is the possibility that curative surgeries were misclassified as palliative, which would tend to enrich the proportions of resective surgeries and seizure freedom. A more standardized definition of epilepsy surgery with palliative intent in future research would help mitigate this potential source of bias. We measured epilepsy duration between the onset of seizures and time of surgery; however, we are not able to account for variation in time to establish drug resistance, which may exceed 4 years in up to one quarter of patients with DRE.^25^ The median follow‐up in our cohort was 21 months; it is possible we may have seen more benefit with longer postprocedure times (e.g., 24, 36, or 48 months), specifically in neuromodulation, where seizure reduction improves over time.^26^ Additionally, 60% of our neuromodulation cohort underwent VNS; per the enrollment criteria, all patients who underwent VNS were evaluated for at least one other procedure. This limitation may bias our VNS population toward more complex cases, resulting in less favorable outcomes. Together, these factors may explain why our study did not reproduce other results in the literature that earlier VNS implantation leads to greater odds of meaningful seizure reduction.^13^, ^14^, ^15^

Finally, we did not consider secondary outcomes such as neurocognitive scores or quality of life metrics. The impact of epilepsy surgery on cognition is complex. Prior work by Skirrow et al. in patients undergoing temporal lobectomy found that although intelligence quotient (IQ) improved after surgery, epilepsy duration was not a significant predictor of postsurgical IQ.^27^ A recent meta‐analysis in patients undergoing RNS did not find any significant differences between pre‐ and postoperative neurocognitive outcomes.^28^ There is also evidence that neuropsychologic testing is underutilized in patients undergoing epilepsy surgery with palliative intent, and potential benefits may not be recognized.^29^ Future work examining the impact of epilepsy surgery with palliative intent and the timing of surgery on neurocognitive outcomes may identify subsets of patients who may benefit in outcomes beyond seizure reduction.

## 5 CONCLUSIONS

We did not find an association between early epilepsy surgery and seizure reduction in our cohort of patients undergoing procedures with palliative intent. This finding reflects the heterogeneity of our population, including many different types of surgical procedures and both lesional and nonlesional epilepsies. We found that procedures with palliative intent generally produce reduction in seizure frequency equal to or better than that expected from additional ASM trials. We also found evidence that early epilepsy surgery is important in some patients undergoing palliative resective surgery. In the future, a standardized definition of what constitutes epilepsy surgery with palliative intent would help improve interrater reliability between centers and may impact the associations found within this study. Future work may elucidate the subpopulation of patients who would most benefit from early surgical intervention, which may require more stringent selection criteria in presurgical evaluation. Furthermore, we found that patients with clinical factors that predisposed to poorer outcome in terms of seizure reduction also had longer times to surgery. Further work should examine other outcome measures such as quality of life and neurocognitive outcomes to ensure that these patients are not deprived of any potential benefits of epilepsy surgery by delaying referral for evaluation.

## AUTHOR CONTRIBUTIONS

**Robert M. Crutcher:** Drafting/revision of the manuscript for content, including medical writing for content; major role in the acquisition of data; study concept or design; analysis or interpretation of data. **David E. Horvat:** Drafting/revision of the manuscript for content, including medical writing for content; major role in the acquisition of data; study concept or design; analysis or interpretation of data. **Avery R. Caraway:** Drafting/revision of the manuscript for content, including medical writing for content; major role in the acquisition of data analysis or interpretation of data. **Kristen H. Arredondo:** Drafting/revision of the manuscript for content, including medical writing for content; major role in the acquisition of data. **Taylor** **J. Abel:** Drafting/revision of the manuscript for content, including medical writing for content; major role in the acquisition of data. **Nitin Agarwal:** Drafting/revision of the manuscript for content, including medical writing for content; major role in the acquisition of data. **Allyson L. Alexander:** Drafting/revision of the manuscript for content, including medical writing for content; major role in the acquisition of data. **Dallas Michael Armstrong:** Drafting/revision of the manuscript for content, including medical writing for content; major role in the acquisition of data. **Kurtis I. Auguste:** Drafting/revision of the manuscript for content, including medical writing for content; major role in the acquisition of data. **Danilo Bernardo:** Drafting/revision of the manuscript for content, including medical writing for content; major role in the acquisition of data. **Jeffrey Bolton:** Drafting/revision of the manuscript for content, including medical writing for content; major role in the acquisition of data. **David Bonda:** Drafting/revision of the manuscript for content, including medical writing for content; major role in the acquisition of data. **Dewi Frances Depositario‐Cabacar:** Drafting/revision of the manuscript for content, including medical writing for content; major role in the acquisition of data. **Krista Eschbach:** Drafting/revision of the manuscript for content, including medical writing for content; major role in the acquisition of data. **William D. Gaillard:** Drafting/revision of the manuscript for content, including medical writing for content; major role in the acquisition of data. **Aran Groves:** Drafting/revision of the manuscript for content, including medical writing for content; major role in the acquisition of data. **Jason S. Hauptman:** Drafting/revision of the manuscript for content, including medical writing for content; major role in the acquisition of data. **Pradeep K. Javarayee:** Drafting/revision of the manuscript for content, including medical writing for content; major role in the acquisition of data. **Cemal Karakas:** Drafting/revision of the manuscript for content, including medical writing for content; major role in the acquisition of data. **Andrew T. Knox:** Drafting/revision of the manuscript for content, including medical writing for content; major role in the acquisition of data. **Jenny J. Lin:** Drafting/revision of the manuscript for content, including medical writing for content; major role in the acquisition of data. **Patricia E. McGoldrick:** Drafting/revision of the manuscript for content, including medical writing for content; major role in the acquisition of data. **Derryl J. Miller:** Drafting/revision of the manuscript for content, including medical writing for content; major role in the acquisition of data. **Edward John Novotny:** Drafting/revision of the manuscript for content, including medical writing for content; major role in the acquisition of data. **Adam P. Ostendorf:** Drafting/revision of the manuscript for content, including medical writing for content; major role in the acquisition of data. **Pilar D. Pichon:** Drafting/revision of the manuscript for content, including medical writing for content; major role in the acquisition of data. **Shilpa B. Reddy:** Drafting/revision of the manuscript for content, including medical writing for content; major role in the acquisition of data. **Debopam Samanta:** Drafting/revision of the manuscript for content, including medical writing for content; major role in the acquisition of data. **Rani K. Singh:** Drafting/revision of the manuscript for content, including medical writing for content; major role in the acquisition of data. **Janelle L. Wagner:** Drafting/revision of the manuscript for content, including medical writing for content; major role in the acquisition of data. **Steven M. Wolf:** Drafting/revision of the manuscript for content, including medical writing for content; major role in the acquisition of data. **Lily C. Wong‐Kisiel:** Drafting/revision of the manuscript for content, including medical writing for content; major role in the acquisition of data. **Fernando N. Galan:** Drafting/revision of the manuscript for content, including medical writing for content; major role in the acquisition of data; study concept or design; analysis or interpretation of data. **M. Scott Perry:** Drafting/revision of the manuscript for content, including medical writing for content; major role in the acquisition of data; study concept or design; analysis or interpretation of data. **Michael A. Ciliberto:** Drafting/revision of the manuscript for content, including medical writing for content; major role in the acquisition of data; study concept or design; analysis or interpretation of data.

## FUNDING INFORMATION

This study received no specific funding.

## CONFLICT OF INTEREST STATEMENT

P.E.M. reports the following conflicts: NeuroPace: advisory board, speaker, pharma‐sponsored study; Eisai: advisory board, speaker, pharma‐sponsored study; Jazz: advisory board, speaker, pharma‐sponsored study, copyright license; LivaNova: advisory board, speaker, pharma‐sponsored study; Aquestive: advisory board, pharma‐sponsored study; Neurelis: advisory board, speaker, pharma‐sponsored study; UCB; advisory board, speaker, pharma‐sponsored study, copyright license; SK Life, pharma‐sponsored study; Longboard: pharma‐sponsored study; Takeda: pharma‐sponsored study; Monteris: advisory board, speaker; Marinus: advisory board, speaker; Biohaven: pharma‐sponsored study; Assertio: advisory board. S.M.W. reports the following conflicts: NeuroPace: advisory board, speaker, pharma‐sponsored study; Eisai: advisory board, speaker, pharma‐sponsored study; Jazz: advisory board, speaker, pharma‐sponsored study, copyright license; LivaNova: advisory board, speaker, pharma‐sponsored study; Aquestive: advisory board, pharma‐sponsored study; Neurelis: advisory board, speaker, pharma‐sponsored study; UCB; advisory board, speaker, pharma‐sponsored study, copyright license; SK Life, pharma‐sponsored study; Longboard: pharma‐sponsored study; Takeda: pharma‐sponsored study; Monteris: advisory board, speaker; Marinus: advisory board, speaker; Biohaven: pharma‐sponsored study; Assertio: Advisory board. F.N.G. reports consulting work for Jazz Pharmaceuticals, Biocodex, UCB, and Stoke. M.S.P. reports honoraria for consulting from UCB, Neurelis, Stoke Therapeutics, Biocodex, Orphalan, and GenKOre; research funding paid to Cook Children's from Stoke Therapeutics and Encoded Therapeutics; and publicly traded stock ownership of Praxis Precision Medicines, Rapport Therapeutics, and CAMP4 Therapeutics. M.A.C. reports salary support from Stoke and Takeda. The views expressed in this article are those of the authors and do not necessarily reflect the official policy or position of the Alexander T. Augusta Military Medical Center, Defense Health Agency, Department of Defense, or US Government. The rest of the authors have no relevant disclosures. We confirm that we have read the Journal's position on issues involved in ethical publication and affirm that this report is consistent with those guidelines.

## Data Availability

The data that support the findings of this study are available on request from the corresponding author. The data are not publicly available due to privacy or ethical restrictions.
